# Entropy-Based Age-Aware Scheduling Strategy for UAV-Assisted IoT Data Transmission

**DOI:** 10.3390/e27060578

**Published:** 2025-05-29

**Authors:** Lulu Jing, Hai Wang, Zhen Qin, Peng Zhu

**Affiliations:** 1 College of Communication Engineering, Army Engineering University of PLA, Nanjing 210000, China; 2 Department of Information and Communication, Noncommissioned Officer Academy of PAP, Hangzhou 310000, China; 3 Academy of Systems Engineering, Academy of Military Sciences, Beijing 100000, China

**Keywords:** unmanned aerial vehicle relay, age of information, restless multi-armed bandit, l-conditional cross-entropy, gain-index-based policy

## Abstract

This paper investigates data transmission in an Internet of Things (IoT) network, where multiple devices send environmental data to a remote base station through an unmanned aerial vehicle (UAV) relay. The UAV serves as an airborne intermediary that collects status information from distributed IoT devices (e.g., temperature readings in a real-time forest fire monitoring system) and forwards it to the base station. To capture the impact of data staleness, a novel Age of Information (AoI) and entropy-aware system loss is defined in terms of L-conditional cross-entropy, which quantifies the expected penalty caused by state misestimation. The scheduling problem, which aims to minimize the system loss defined by L-conditional cross-entropy, is formulated as a Restless Multi-Armed Bandit (RMAB) problem. By applying Lagrange relaxation, the objective function is decomposed into tractable sub-problems, enabling a low-complexity, gain-index-based scheduling strategy. Numerical simulations validate the effectiveness of the proposed algorithm in reducing the long-term average system loss. In particular, the gain-index-based policy achieves a significant reduction in average penalty compared to random, round-robin, periodic update, and MAX-AoI scheduling strategies, demonstrating its superior performance over these baselines.

## 1. Introduction

Since its inception, the Internet of Things (IoT) has garnered significant attention and found widespread applications across diverse domains, including industrial manufacturing, intelligent transportation, healthcare, environmental monitoring, and military security [1,2]. Architecturally, an IoT system typically comprises three core components: IoT devices, transmission networks, and base stations (BS). IoT devices, often referred to as sources, are frequently deployed in remote and challenging environments for the purpose of capturing physical attributes of their surroundings, such as temperature, humidity, and pollution levels. However, inherent limitations in energy and transmission power impede these devices’ ability to establish long-range communication. To address this constraint and enhance data transmission efficiency in ground-based IoT systems, Unmanned Aerial Vehicles (UAVs) have been adopted as aerial relays, facilitating the conveyance of sensed data from geographically dispersed IoT devices to centralized ground base stations [3]. At the base station, the received data, which may include parameters like temperature, humidity, or carbon monoxide concentration in the context of a forest fire monitoring system, undergoes processing to extract meaningful insights that inform decision-making processes. Critically, the accuracy and timeliness of such decisions heavily depend on the freshness of the extracted information.

The seminal work by Kaul et al. [4] introduced the Age of Information (AoI) as a metric to quantify the freshness of information in communication networks. Since then, AoI has received extensive attention across various network paradigms, including remote monitoring, edge computing, and time-sensitive applications [5,6,7,8,9,10,11,12,13,14,15]. Several studies [6,9,11] have focused on minimizing the average AoI across all IoT nodes in UAV-assisted data collection systems. These studies have demonstrated that carefully designed UAV trajectory optimization strategies—such as the selection of optimal hovering locations and the determination of efficient access sequences—can significantly reduce the overall AoI in UAV-assisted IoT systems. In scenarios involving unknown traffic patterns, Markov Decision Process (MDP) models have been adopted to guide scheduling policies that reduce long-term AoI [10]. In addition to trajectory optimization, numerous centralized scheduling strategies have been proposed to coordinate UAV-based data collection. For instance, the UAV adopts the MAF-MAD strategy, applying Maximum AoI First for sampling IoT devices (Hop 1) and Maximum AoI Difference for forwarding data to the base station (Hop 2), thereby prioritizing updates with the highest freshness gap [12]. Building upon this, [13] proposed an airborne cooperative relaying and AoI-sensitive data collection (ADC) framework, which iteratively optimizes UAV flight paths using a generalized AoI expectation function. The problem has also been extended to multi-UAV scenarios, where joint optimization of trajectories and scheduling is required to minimize system-wide AoI. For example, [14] formulates a multi-stage stochastic optimization problem that jointly manages UAV data buffers and AoI states through queueing models, and proposes an adaptive, age-aware scheme for multi-UAV deployments. Recent research on UAV-assisted IoT systems has largely focused on minimizing freshness metrics such as the AoI. These efforts reflect a growing recognition that simply reducing AoI is insufficient to capture the true value of the monitored data, especially in UAV-assisted IoT systems where timely and informative updates are critical for accurate decision-making.

## 2. Related Work

To address the limitations of conventional AoI, researchers have proposed a range of enhanced metrics aimed at capturing more nuanced aspects of information freshness and utility. For instance, the Age of Incorrect Information (AoII) [16] integrates both AoI and estimation error to more accurately reflect the impact of outdated data on system performance. In [17], the authors proposed a semi-Markov decision framework for optimizing remote estimation with threshold-based updates triggered by the AoII. They introduced a dual-regime absorbing Markov chain model to characterize AoII distributions and minimize a weighted sum of AoII and transmission costs, outperforming benchmark strategies in simulations. In [18], the Age of Synchronization (AoS) is combined with AoI to optimize refresh rate allocation in multi-source systems under bandwidth constraints. Further advancing this line of research, [19] introduces the Utility of Information (UoI), which explicitly accounts for the nonlinear, time-varying importance, and context-dependent value of status updates. Despite these advancements, existing metrics still fall short in fully capturing the interaction between the underlying information structure of the source and the dynamic estimation state at the receiver, thereby limiting their effectiveness in supporting precise remote estimation. In [20], a context-aware status updating framework was proposed for multi-sensor systems in safety-critical environments. The authors designed a penalty function sensitive to both AoI and signal value, and formulated a pull-based scheduling problem as a Restless Multi-Armed Bandit (RMAB), which they solved via Lagrange relaxation to derive a low-complexity, asymptotically optimal policy. In [21], the authors proposed a semantics-aware remote estimation framework for discrete-state Markov sources with normal and alarm states. They introduced two novel metrics, the Age of Missed Alarm (AoMA) and the Age of False Alarm (AoFA), to capture the asymmetric costs and lasting impacts of different estimation errors. The scheduling problem was formulated as an infinite-state Markov decision process (MDP), and the optimal policy was shown to follow a threshold-based switching structure. In [22], the authors proposed a semantics-aware remote estimation framework with a new metric, Age of Consecutive Error (AoCE), capturing both the severity and persistence of estimation errors, and derived structured transmission policies via MDP modeling. In [23], the authors studied a multi-sensor system in which each sensor monitors multiple correlated information processes and transmits updates via a shared channel. They analyzed the impact of correlation on both average AoI and state estimation errors, optimized sensing resource allocation under limited capabilities, and highlighted critical thresholds where optimal strategies shift abruptly.

Despite these advances, existing AoI-based metrics fail to capture performance degradation in remote monitoring systems from an information-theoretic perspective. In particular, they do not leverage entropy to quantify the system performance loss caused by information staleness. This gap motivates this present work, which introduces an entropy-based loss formulation tailored for UAV-assisted IoT scheduling, and develops a tractable gain-index policy to minimize such loss.

Motivated by these limitations, this paper proposes a novel information-theoretic formulation that quantifies the system performance degradation caused by information staleness. The system loss is defined using an L-conditional cross-entropy framework. This metric reflects the expected penalty caused by state prediction errors due to outdated updates at the receiver. This formulation allows for a more accurate assessment of the impact of outdated information. Integrated into a scheduling framework, it aims to minimize long-term average penalty, thereby enhancing both estimation accuracy and decision-making. The main contributions are summarized as follows:

1. The limitations of Age of Information (AoI) as a performance metric in remote state estimation are first critically analyzed. To address these shortcomings, we conduct an entropy-based analysis of the impact of AoI on system performance. Specifically, we introduce the concept of L-conditional cross-entropy and propose a novel entropy-based, AoI-aware loss formulation that provides a more accurate quantification of system performance degradation caused by outdated information.
2. Building on this insight, a novel UAV-assisted monitoring framework is developed, incorporating a generalized, AoI-induced, state-dependent loss function in which performance degradation caused by stale information is modeled using an L-conditional cross-entropy formulation. Unlike conventional conditional entropy, which quantifies the residual uncertainty in the true state given an estimate, L-conditional cross-entropy directly models the expected loss resulting from estimation errors under outdated data. This work pioneers an information-theoretic integration of semantic entropy quantification into AoI-driven scheduling for UAV-assisted IoT networks, establishing a novel framework that quantifies information uncertainty through entropy measures while optimizing data freshness. Unlike conventional approaches, our model explicitly incorporates differential misclassification costs through L-conditional cross-entropy—particularly critical in safety-sensitive scenarios where information value asymmetry exists (e.g., the loss incurred from misclassifying a “Fire” state as “Normal” is significantly greater than that from the reverse).
3. Based on a system level loss formulation, an innovative penalty-based gain function is constructed, along with a theoretical lower bound for system performance using the concept of L-conditional cross-entropy. The status update scheduling problem in pull-based UAV-IoT systems is then modeled as a Restless Multi-Armed Bandit (RMAB) problem. By applying Lagrange relaxation with dual decomposition, the global objective is decomposed into computationally tractable single-device subproblems. The convergence of the dual problem is guaranteed by the diminishing step size rule $\alpha_{t}=\frac{c}{t}$, and the convexity of the dual function, as established in Appendix A. Unlike Whittle’s index policy, which requires indexability assumptions, the proposed gain-index-based strategy introduces a novel index construction that eliminates this requirement, thereby significantly enhancing its applicability to heterogeneous and dynamically evolving environments commonly found in UAV-assisted IoT networks.
4. The effectiveness of the proposed novel gain-index-based scheduling strategy is validated through extensive numerical simulations. The evaluation, conducted under both uniform and weighted node configurations, demonstrates for the first time that incorporating entropy-based penalties into AoI-based UAV scheduling significantly improves performance in minimizing long-term average system loss. The proposed method consistently outperforms conventional baselines—including Random, Round-Robin, Periodic Update and MAX-AoI schedule policies—thereby demonstrating the broad effectiveness of the strategy.

The remainder of this paper is organized as follows: Section 2 reviews related work on AoI-aware scheduling, semantic utility metrics, and RMAB-based policies. Section 3 presents the UAV-assisted IoT system model and introduces the L-conditional cross-entropy-based loss formulation. Section 4 formulates the scheduling problem as a Restless Multi-Armed Bandit and derives a gain-index-based policy via Lagrange relaxation. Section 5 provides simulation results comparing the proposed strategy with several baselines. Section 6 concludes the paper and outlines potential directions for future work. Section 7 provides a summary of this paper.

## 3. System Model and Methodology

### 3.1. UAV-Relayed IoT Remote Monitoring System

**Figure 1:**
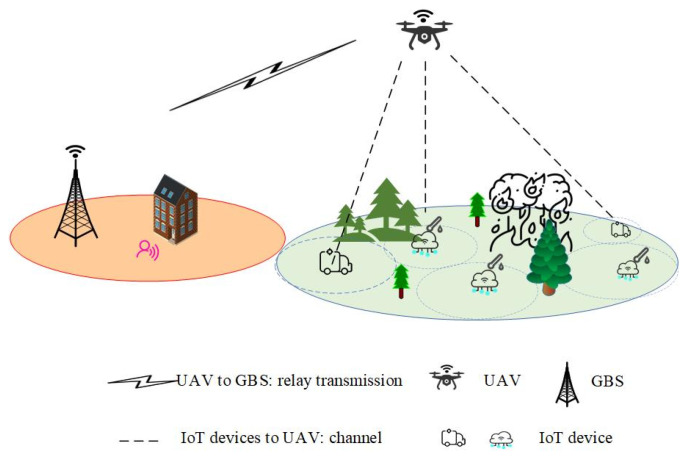
UAV-relayed IoT device status updating system.

This paper considers a pull-based update mechanism, in which the GBS only requests status updates from IoT devices through a UAV relay when it is uncertain about the system state. Under this mechanism, we consider the status updating system illustrated in Figure 1, where a UAV is deployed as a fixed aerial base station, providing end-to-end wireless coverage between the ground base station (GBS) and IoT devices located within the communication range of the UAV relay. Due to communication limitations—such as the restricted transmission power of IoT devices, signal attenuation caused by physical obstacles (e.g., dense vegetation, buildings, and terrain irregularities), and the limited communication range inherent in low-power wireless technologies (such as ZigBee or Wi-Fi)—direct and reliable communication between IoT devices and the ground base station (GBS) is often challenging or infeasible. For example, in forest fire monitoring scenarios, dense vegetation and uneven terrain significantly weaken signals from devices, making it difficult for them to reliably transmit data directly to distant ground stations. Therefore, the UAV relay acts as an intermediary, facilitating communication between the GBS and IoT devices. In the monitoring area, N IoT devices transmit crucial status updates to the UAV through M wireless channels.

Let T denote the total observation duration, which is divided into multiple time slots of equal length, denoted by t=0,1,2⋯⋯T−1. Each device n∈N generates Markovian state information $X_{n,t}\in X$ for the remote monitoring system. $X_{n,t}$ may represent information related to forest fire status, such as temperature, humidity, smoke concentration, and atmospheric quality. We use to quantify the hazard level of the fire-monitoring system, for example, Normal, Alert, or Fire, where $Y_{n,t}$ is a function of the system state $X_{n,t}$. In response to a pull request, each device n generates and submits a time-stamped updating message ($X_{n,t},t$) to one wireless channel. We assume that transmitting an update message to the receiver requires one time slot. However, the transmission of status updates is unreliable due to the inherent fading characteristics of wireless channels. Let $l_{n}$ denote the probability of successful transmission from device n to the UAV, and let $l_{r}$ denote the probability of successful transmission from the UAV to the GBS, which is independent of the chosen wireless channel.

We focus on the AoI at the GBS, denoted by $\Delta \left(t\right)\in \mathbb{N}$, which is defined as the time elapsed since the generation of the most recently received status update. Due to the unreliability of the channel state, the data received by the GBS will be stale. We represent this data by $X_{n,t-\Delta \left(t\right)}$, which was generated $\Delta \left(t\right)$ time slots ago. At each time slot t, the information age evolution of the status update of IoT device *n* is given by the following:

(1) $$\Delta_{n}\left(t+1\right)=\left\{\begin{array}{l} \Delta_{n}\left(t\right)+1\;\mathrm{with}\;\mathrm{probability}\;1-l_{n}\times l_{r}\;\\ 1\;\;\;\;\;\;\;\;\;\;\;\;\;\;\;\;\mathrm{with}\;\mathrm{probability}\;l_{n}\times l_{r} \end{array}\right.,$$

Specifically, at each time slot t, if the transmission from IoT device n to the UAV and from the UAV to the GBS are both successful (with a combined probability $l_{n}\times l_{r}$), the AoI for device n resets to 1. Otherwise, the AoI $\Delta \left(t\right)$ increments by 1, indicating that the received information is becoming older. This reflects how communication reliability directly impacts information freshness at the base station.

### 3.2. Loss Model for UAV-Relayed IoT Remote Monitoring System

Consider the UAV-relayed IoT remote monitoring system shown in Figure 1, which provides real-time hazard level estimations through monitoring data provided by various IoT devices. For example, in a fire detection system, the latest state information of IoT device n is $X_{n,t-\Delta \left(t\right)}$, and the system estimates the hazard level as $o=\phi \left(X_{n,t-\Delta \left(t\right)},\Delta \left(t\right)\right)\in O$, where ϕ is a function of $X_{n,t-\Delta \left(t\right)}$ and $\Delta \left(t\right)$. Inherently, errors exist in estimating the hazard level associated with the monitored area’s state. These estimation errors are characterized by a loss function $L\left(Y_{n,t},o\right)$, which quantifies the difference between the estimated and actual values, providing a measure of model performance.

Building upon the concept of remote monitoring, consider a fire detection system capable of assessing fire risk in specific areas. The system uses sensor data to track the fire status in real-time. However, it is important to note that the estimated state produced by the monitoring system may not always match the actual fire state. This misestimation could have significant implications, such as delayed responses or unnecessary actions based on inaccurate fire detection.

The loss function values, as shown in Table 1, are used to quantify the errors in the system’s state estimations. For instance, if the system estimates the state hazard level as *Normal* when there is a Fire (i.e., the true state is Fire, and the estimated state is Normal), the loss function $L\left(F,N\right)=200$, indicating a significant misclassification that could lead to disastrous consequences.

It is important to note that the loss values in Table 1 do not represent actual economic losses. Instead, they serve as theoretical penalty terms defined for modeling purposes, aiming to reflect the relative severity of different misclassification outcomes within the system.

To reflect the real-world costs of different types of misclassifications (e.g., falsely estimating a Fire state as Normal), a penalty structure is designed in which the severity of each error is encoded using asymmetric values. For example, minor misjudgments (e.g., mistaking Alert for Normal) are assigned a penalty of 50, while severe misclassifications (e.g., predicting Fire as Normal) are assigned 300 or more, reflecting at least a 6-fold increase in consequence. These values are not intended to represent exact monetary losses, but to capture the relative severity of different errors.

### 3.3. Information-Theoretic Metric for UAV-Relayed IoT Remote Monitoring System

In a UAV-relayed IoT remote monitoring system, the estimated loss in system performance resulting from an IoT device’s action in a given state $\left(X_{n,t-\Delta \left(t\right)},\Delta \left(t\right)\right)$ at time slot t is defined by the following penalty function:

(2) $$p_{n}\left(x,\delta \right)=E\left[L\left(Y_{n,t},o\right)\left|\left(X_{n,t-\Delta \left(t\right)}=x,\Delta \left(t\right)=\delta \right)\right.\right],$$

where $o=\phi \left(X_{n,t-\Delta \left(t\right)},\Delta \left(t\right)\right)\in O$ is the estimated output of the state hazard level, ϕ is any function that maps from X×ℕ to O. Consider the optimization task described below:

(3) $$\underset{o\in O}{\min}E\left[L\left(Y_{n,t},o\right)\left|\left(X_{n,t-\Delta \left(t\right)}=x,\Delta \left(t\right)=\delta \right)\right.\right],$$

Let ϕ∗ be the optimal estimator solving the estimation problem in Equation (3). Then, we denote its output using the following

(4) $$o^{*}=\phi^{*}\left(X_{n,t-\Delta \left(t\right)},\Delta \left(t\right)\right),$$

After substituting this optimal estimator into Equation (2), we obtain the following:

(5) $$p_{n}\left(x,\delta \right)=E\left[L\left(Y_{n,t},\phi *\left(X_{n,t-\Delta \left(t\right)},\Delta \left(t\right)\right)\right)\left|X_{n,t-\Delta \left(t\right)}=x,\Delta \left(t\right)=\delta \right.\right],$$

This equation represents the minimum achievable expected loss, meaning that for every estimator o∈O, the penalty cannot be lower than this bound. As stated in [24], the penalty function in Equation (5) is intrinsically linked to the concept of entropy. Let $o={\hat{Y}}_{n,t}$ be the estimation of $Y_{n,t}$. Subsequently, the L-cross entropy and L-conditional cross-entropy between $Y_{n,t}$ and ${\hat{Y}}_{n,t}$ are defined.

(6) $$H_{L}\left(Y_{n,t};{\hat{Y}}_{n,t}\right)=E\left[L\left(Y_{n,t},o\right)\right],$$

Equation (6) defines the L-cross entropy, which means the penalty caused by the difference between $Y_{n,t}$ and ${\hat{Y}}_{n,t}$

(7) $$H_{L}\left(Y_{n,t};{\hat{Y}}_{n,t}\left|X_{n,t-\Delta \left(t\right)}=x,\Delta \left(t\right)=\delta \right.\right)=E\left[L\left(Y_{n,t},o\right)\left|X_{n,t-\Delta \left(t\right)}=x,\Delta \left(t\right)=\delta \right.\right],$$

Intuitively, the L-conditional cross-entropy in Equation (7) quantifies the expected loss caused by estimation errors under stale information, especially when the loss function is asymmetric and task-specific. Equation (7) formally defines this quantity, measuring the anticipated loss due to the divergence between $Y_{n,t}$ and ${\hat{Y}}_{n,t}$, conditional on the observed variables $X_{n,t-\Delta \left(t\right)}$ and $\Delta \left(t\right)$. Minimizing $H_{L}\left(\cdot \right)$ directly encourages the estimated state to align more closely with the true state, as the optimal estimator forces ${\hat{Y}}_{n,t}\to Y_{n,t}$.

Consequently, this reduction in penalty due to estimation errors manifests as a lower entropy value, which improves the estimation accuracy of remote monitoring systems by suppressing error propagation caused by suboptimal state estimation.

### 3.4. L-Conditional Cross-Entropy and Its Lower Bounds

Based on the L-conditional cross-entropy definition, Equation (7) formulates the expected loss measure conditioned on a given estimator o∈O. Minimizing this quantity is more challenging than directly computing the L-conditional entropy. Using Gibbs’ inequality [25] and Jensen’s Inequality [26], the L-conditional entropy is proven to be the theoretical lower bound, as formally expressed by:

(8) $$H_{L}\left(Y_{n,t}\left|X_{n,t-\Delta \left(t\right)}=x,\Delta \left(t\right)=\delta \right.\right)\leq H_{L}\left(Y_{n,t};{\hat{Y}}_{n,t}\left|X_{n,t-\Delta \left(t\right)}=x,\Delta \left(t\right)=\delta \right.\right).$$

To enforce this bound, we reformulate the original optimization problem (3) which minimizes the penalty in Equation (2). By integrating the theoretical insights from Equation (8), the original problem is transformed into the following formulation:

(9) $$\begin{array}{c} H_{L}\left(Y_{n,t}\left|X_{n,t-\Delta \left(t\right)}=x,\Delta \left(t\right)=\delta \right.\right)=\underset{o\in O}{\min}H_{L}\left(Y_{n,t};{\hat{Y}}_{n,t}\left|X_{n,t-\Delta \left(t\right)}=x,\Delta \left(t\right)=\delta \right.\right)\\ \;\;\;\;\;\;\;\;\;\;\;\;\;\;\;\;\;\;\;\;\;\;\;\;\;\;\;\;\;\;\;\;\;\;\;\;\;\;\;\;\;\;\;\;\;\;\;\;\;\;\;\;\;\;=\underset{o\in O}{\min}E\left[L\left(Y_{n,t},o\right)\left|X_{n,t-\Delta \left(t\right)}=x,\Delta \left(t\right)=\delta \right.\right]\\ \;\;\;\;\;\;\;\;\;\;\;\;\;\;\;\;\;\;\;\;\;\;\;\;\;\;\;\;\;\;\;\;\;\;\;\;\;\;\;\;\;\;\;\;\;\;\;\;\;=E\left[L\left(Y_{n,t},o^{*}\right)\left|X_{n,t-\Delta \left(t\right)}=x,\Delta \left(t\right)=\delta \right.\right] \end{array},$$

From Equations (5) and (9), it is evident that

(10) $$p_{n}\left(x,\delta \right)=H_{L}\left(Y_{n,t}\left|X_{n,t-\Delta \left(t\right)}=x,\Delta \left(t\right)=\delta \right.\right).$$

For the optimal estimator $o^{*}$, the expected loss is indeed the L-conditional entropy, which is an information-theoretic lower bound of $p_{n}\left(x,\delta \right)$. This bound defines the fundamental performance limit by quantifying the performance degradation caused by incomplete status awareness. Notably, existing metrics proposed in prior studies—such as AoII, UoI and AoS—fail to characterize this information-theoretic limit.

### 3.5. Penalty Function and AoI

**Figure 2:**
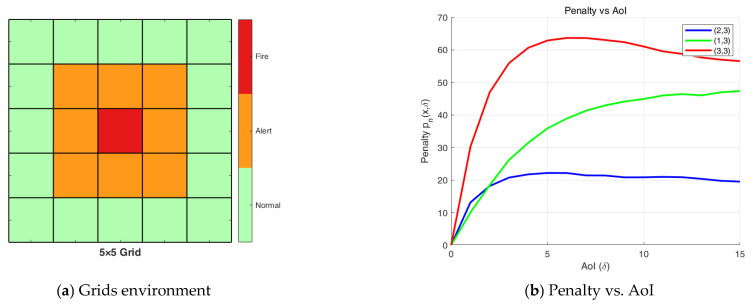
(**a**) Grids based on different fire hazard levels; (**b**) Penalty vs. AoI for different initial states.

As previously stated, we consider a remote real-time fire monitoring system, where the fire hazard level is classified into three categories: Normal, Alert, or Fire. The monitoring area is divided into grids based on three levels, and N IoT devices are deployed accordingly. In this structure, the grid center at position (3,3) is designated as Fire, the immediate surrounding cells form the *Alert* region, and the outermost layer constitutes the Normal region. Upon each pull request, device n reports a state vector $X_{n,t}$, which captures the spatial distribution trend of local temperature, particularly under delayed updates. We model temperature evolution as a stochastic process, where the state remains unchanged with probability 0.7 and transitions (e.g., rising or falling) occur with probability 0.3. The resulting spatial patterns under delayed updates are illustrated in Figure 2a. Our analytical results demonstrate that the penalty function $p_{n}\left(x,\delta \right)$ exhibits non-monotonic behavior with respect to the Age of Information with different initial states, as shown in Figure 2b.

From Figure 2b, we observe that regions closer to the Fire area exhibit a more rapid increase in penalty due to the higher loss value associated with their state transitions in subsequent time slots. Moreover, the penalty curve does not necessarily vary monotonically with increasing AoI. This non-monotonic behavior arises because the penalty reflects the expected loss under information staleness—not just the AoI itself. As such, commonly used metrics like AoI, AoII, and UoI fail to fully capture this behavior.

### 3.6. Problem Formulation

Due to transmission errors and limited communication resources, device updates may not be timely, leading to misunderstandings of the current situation. To minimize the penalty, we study a pull-based multi-channel transmission scheduling problem, where a central scheduler actively selects which devices to query at each time slot in order to minimize prediction loss. Unlike push-based approaches, where devices autonomously transmit their updates regardless of system needs, the pull-based strategy allows the scheduler to selectively request updates based on each device’s current information age or potential impact on system performance. This helps prioritize critical updates and reduce redundant communication overhead. The scheduling policy is denoted by $\pi =\left\{\pi_{1},\pi_{2},\cdots \pi_{N}\right\}$, where $\pi_{n}=\left(u_{n}\left(0\right),u_{n}\left(1\right),\cdots \right)$ determines whether status information from device n is scheduled for transmission at each time slot t. Let Π be the set of all scheduling policies and π∈Π. Since status updates from the N IoT devices are transmitted over M shared channels, the constraint $\sum_{n=1}^{N}u_{n}\left(t\right)\leq M$ must be satisfied.

Our objective is to identify an optimal scheduling policy that minimizes the total expected loss across all N IoT devices over a finite time horizon T. Accordingly, the time-averaged scheduling optimization problem can be formulated as follows:

(11) $${\bar{p}}_{opt}=\underset{\pi \in \Pi }{\inf}\underset{T\to \infty }{\lim\sup}\sum_{n=1}^{N}E_{\pi }\left[\frac{1}{T}\sum_{t=0}^{T-1}\omega_{n}p_{n}\left(X_{n,t-\Delta \left(t\right)},\Delta \left(t\right)\right)\right],$$

(12) $$s.t.\sum_{n=1}^{N}u_{n}\left(t\right)\leq M,u_{n}\left(t\right)\in \left\{0,1\right\},\forall t,$$

where $p_{n}\left(X_{n,t-\Delta \left(t\right)},\Delta \left(t\right)\right)$ is the penalty incurred by device n at slot t as defined in Equation (2), $\omega_{n}$ is the weight value of device n, and ${\bar{p}}_{opt}$ represents the optimal value of problem (11). Equation (12) specifies the scheduling constraints, ensuring that at most M devices are selected for transmission at each time slot t.

## 4. The Scheduling Problem and Policy

Due to the channel resource constraint in problem (11)–(12), when N>M, not all devices can transmit updates simultaneously in each time slot. To address this, we develop an efficient scheduling policy that minimizes the time-averaged total expected penalty across all N devices while strictly satisfying constraint (12). The detailed design is presented in the following section.

### 4.1. RMAB Formulation

The scheduling problem can be formulated as an RMAB. Specifically, each device n is an arm and $\left(X_{n,t-\Delta \left(t\right)},\Delta \left(t\right)\right)$ is the state of each arm. At every time step, we need to allocate the communication channels to M out of N arms in a way that minimizes the sum expected penalty of all arms. The formal definition of this RMAB problem is presented below. Let $u_{n}\left(t\right)\in \left\{0,1\right\}$ denote the action applied to device n at time t, where $u_{n}\left(t\right)=1$ means that n is selected to update in slot t, and $u_{n}\left(t\right)=0$ otherwise. The RMAB problem is generally intractable to solve optimally. While the Whittle index policy is known to be asymptotically optimal for many RMAB problems [27], it requires the satisfaction of a complex condition known as indexability. In this paper, we adopt a low-complexity gain-index policy that avoids such requirements.

### 4.2. Relaxation and Decomposition

Applying the conventional relaxation and Lagrange decomposition approach for RMAB problems [28], we apply the method of Lagrange multipliers to decouple the problem into N single-device sub-problems. The scheduling problem (11)–(12) is then relaxed as follows:

(13) $${\bar{p}}_{opt}=\underset{\pi \in \Pi }{\inf}\underset{T\to \infty }{\lim\sup}\sum_{n=1}^{N}E_{\pi }\left[\frac{1}{T}\sum_{t=0}^{T-1}\omega_{n}p_{n}\left(X_{n,t-\Delta \left(t\right)},\Delta \left(t\right)\right)\right],$$

(14) $$s.t.\underset{T\to \infty }{\lim\sup}\sum_{n=1}^{N}E_{\pi }\left[\frac{1}{T}\sum_{t=0}^{T-1}u_{n}\left(t\right)\right]\leq M,$$

Clearly, constraint (14) provides a relaxed form of the original constraint (12), requiring only average compliance over time, in contrast to Equation (12), which must be satisfied at every time slot t. Based on the relaxation in Equation (14), we employ the Lagrange multiplier method [29] to convert the problem into an unconstrained form by introducing a dual variable λ:

(15) $$\underset{\lambda \geq 0}{\sup}J\left(\lambda \right),$$

(16) $$J\left(\lambda \right)=\underset{\pi \in \Pi }{\inf}\underset{T\to \infty }{\lim\sup}\sum_{n=1}^{N}E_{\pi }\left[\frac{1}{T}\sum_{t=0}^{T-1}\omega_{n}p_{n}\left(X_{n,t-\Delta \left(t\right)},\Delta \left(t\right)\right)+\lambda u_{n}\left(t\right)\right]-\lambda M.$$

Equations (15) and (16) define the dual function $J\left(\lambda \right)$, derived by relaxing the original constrained problem using the Lagrange method. Here, the constraint in Equation (12) is incorporated into the objective using a dual variable λ, resulting in a penalty term λM. For a fixed λ, the term λM is a constant and independent of the scheduling policy π. Thus, the problem in Equation (15) can be decoupled into N independent sub-problems, where each sub-problem corresponding to device n is formulated as follows:

(17) $$J_{n}\left(\lambda \right)=\underset{\pi_{n}\in \Pi_{n}}{\inf}\underset{T\to \infty }{\lim\sup}E_{\pi_{n}}\left[\frac{1}{T}\sum_{t=0}^{T-1}\omega_{n}p_{n}\left(X_{n,t-\Delta \left(t\right)},\Delta \left(t\right)\right)+\lambda u_{n}\left(t\right)\right].$$

where $\pi_{n}=\left(u_{n}\left(0\right),u_{n}\left(1\right),\cdots \right)$ denotes a sub-scheduling policy for devices n, and $\Pi_{\pi }$ represents the set of all admissible policies for the sub-problems $J_{n}\left(\lambda \right)$.

### 4.3. The Gain-Index Policy

To address the decoupled sub-problems described in Equation (17), we propose a low-complexity scheduling policy based on gain indices. The derivation of the gain index follows the index policy construction framework introduced in [30]. Each sub-problem can be modeled as an MDP, where the state is defined as $\left(X_{n,t-\Delta \left(t\right)}=x,\Delta \left(t\right)=\delta \right)$. The Bellman optimality equation under the average cost criterion for the MDP in Equation (17) is given by the following:

(18) $$V_{n}\left(x,\delta ,\lambda \right)+J_{n}\left(\lambda \right)=\min Q_{n}\left(x,\delta ,\lambda ,u\right),$$

where $V_{n}\left(x,\delta ,\lambda \right)$ is the relative-value function and $Q_{n}\left(x,\delta ,\lambda ,u\right)$ is the action value function defined as follows:

(19) $$Q_{n}\left(x,\delta ,\lambda ,u\right)=\omega_{n}p_{n}\left(x,\delta \right)+\lambda u+E\left[V_{n}\left(x^{\prime },\delta^{\prime },\lambda \right)\left|x,\delta ,u\right.\right],$$

It can also be written as the following

(20) $$Q_{n}\left(x,\delta ,\lambda ,u\right)=\left\{\begin{array}{l} \omega_{n}p_{n}\left(x,\delta \right)+V_{n}\left(x,\delta +1,\lambda \right),u=0\\ \omega_{n}p_{n}\left(x,\delta \right)+\lambda +\left(1-\beta_{n}\right)V_{n}\left(x,\delta +1,\lambda \right)+\beta_{n}E\left[V_{n}\left(X_{n,t-1},1,\lambda \right)\left|X_{n,t-\Delta \left(t\right)}=x,\Delta \left(t\right)=1\right.\right],u=1 \end{array}\right.$$

where $\beta_{n}=l_{n}\times l_{r}$. In the context that λ is fixed, we may also simply write the value function as $V_{n}\left(x,\delta \right)$.

We propose the following index policy for the original RMAB problem.

**Definition** **1.** *(Gain Index Policy): For each device* n∈N *, we define a gain index as follows:*

(21) $$g_{n}\left(x,\delta ,\lambda \right)=Q_{n}\left(x,\delta ,\lambda ,1\right)-Q_{n}\left(x,\delta ,\lambda ,0\right),$$

*In each time slot* t *, the gain index* $g_{n}$ *is computed for each device* n*. Based on these indices, the scheduling policy selects the top* M *devices with the highest gain values to transmit their status updates.*

Substituting the definitions from Equation (20), we obtain the following:

(22) $$g_{n}\left(x,\delta ,\lambda \right)=\lambda -\beta_{n}V_{n}\left(x,\delta +1,\lambda \right)+\beta_{n}E\left[V_{n}\left(X_{n,t-1},1,\lambda \right)\left|X_{n,t-\Delta \left(t\right)}=x,\Delta \left(t\right)=1\right.\right],$$

Then, we can obtain the optimal decision for all devices at time t as follows:

(23) $$u_{n}\left(t\right)=\arg\max g_{n}\left(x,\delta ,\lambda \right),$$

We can use a dual subgradient method to solve this problem. For λ, we define the following iteration:

(24) $$\lambda_{t+1}=\max\left\{\lambda_{t}+\alpha_{t}\left(\sum_{n=1}^{N}u_{n}\left(t\right)-M\right),0\right\},$$

Let $\lambda_{0}=0$, $\lambda^{*}$ be the optimal solution to Equation (15), and let $\alpha_{t}$ be the step size. The iterative process stops when the condition $\left|\lambda_{t+1}-\lambda_{t}\right|<\varepsilon$ is satisfied.

Based on Definition 1, we develop a computationally efficient algorithm to solve the relaxed optimization problem defined in Equation (15). The core idea of this approach is the introduction of a gain index value, which serves as a decision metric to guide the selection of scheduling actions $u_{n}\left(t\right)$ for each IoT device. The complete procedure is presented in Algorithm 1 in the form of pseudocode, which outlines the step-by-step execution of the proposed scheduling strategy.
**Algorithm 1** The Gain Index Policy1: **Input:**2:          - Optimal dual variable $\lambda^{*}$3:          - Channel count M, devices N, stepsize $\alpha_{t}$4:          - Device states ($X_{n,t-\Delta_{n}\left(t\right)},\Delta_{n}\left(t\right)$) for all n∈N5: **Output:**6:          - Scheduling policy $u_{n}\left(t\right)\in \left\{0,1\right\}$ for all n∈N7: **Initialization:**8:          - $\lambda_{0}=0$9: **Online Scheduling (for each time slot** t**):**10: **for** each device n=1 to N **do**11:          Update current state ($X_{n,t-\Delta_{n}\left(t\right)},\Delta_{n}\left(t\right)$)12:          Compute gain index $g_{n}\left(x,\delta ,\lambda \right)=Q_{n}\left(x,\delta ,\lambda ,1\right)-Q_{n}\left(x,\delta ,\lambda ,0\right)$ use Equations (18)–(22):13:          Select top M devices with largest positive gain index values use Equation (23)14:          Set $u_{n}\left(t\right)=1$ for selected devices, 0 otherwise>15:          Update $\lambda_{t+1}$ use Equation (24)16: **end for**

In dual-based optimization, it is typically difficult to establish pointwise convergence of the dual iterates or the corresponding function values, especially in the absence of strong convexity or uniqueness. Instead, it is standard to focus on the time-averaged convergence, which can be rigorously derived using projection subgradient analysis. This part of the proof is provided in Appendix A.

## 5. Results

This section presents simulation results to demonstrate the performance of the proposed gain-index policy. We compare the gain index policy, randomized policy (if M channel resources are available, this policy randomly selects at most M devices.), round-robin policy (schedules the devices in sequence according to certain orders and rules), periodic updating (each sensor generates status updates at every time slot and stores them in a first-in-first-out (FIFO) queue. When a communication channel becomes available, the sensor transmits the earliest pending update from the queue), and MAX-AoI (at each time slot t, the MAX-AoI strategy selects the M sensors with the largest Age of Information (AoI) values for transmission).

In the numerical simulation experiment, we consider the square grid area in Figure 2, where the center of the grid is the fire point and the fire spreads outwards. Deployed IoT devices monitor environmental parameters and generate status updates when requested. During observation horizon T→∞, communications occur through a two-hop network of M=5 independent and identically distributed erasure channels, where the first hop involves device-to-UAV transmission with success probability $l_{n}=0.95$, and the second hop (UAV-to-GBS) has a transmission success probability $l_{r}=0.95$. In the iterative process of solving $\lambda^{*}$, we assume that $\varepsilon =10^{-3}$, $\delta_{\max}=T$.

**Figure 3:**
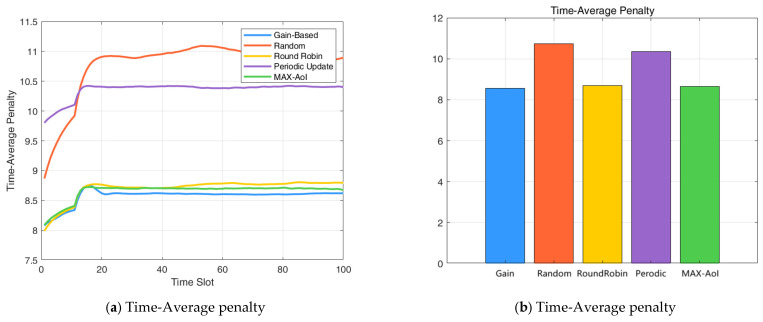
(**a**) Time-Average penalty; (**b**) Time-Average penalty presented as a bar chart.

The performance comparison of the three scheduling policies is presented in Figure 3. The normalized average penalty shown in the figure is computed by dividing the time-averaged system cost by the number of devices. Numerical results indicate that the proposed gain-index-based scheduling policy achieves a notable reduction in average penalty compared to the baseline strategies. In particular, it outperforms the random scheduling policy by 20.33% and the round-robin policy by 1.48%, in terms of long-term average system penalty. Moreover, the gain-index strategy achieves a 17.27% reduction in average penalty compared to the periodic update policy, and a 0.99% reduction compared to the MAX-AoI strategy. These results demonstrate the effectiveness of the proposed method in improving scheduling efficiency under various baseline settings.

**Figure 4:**
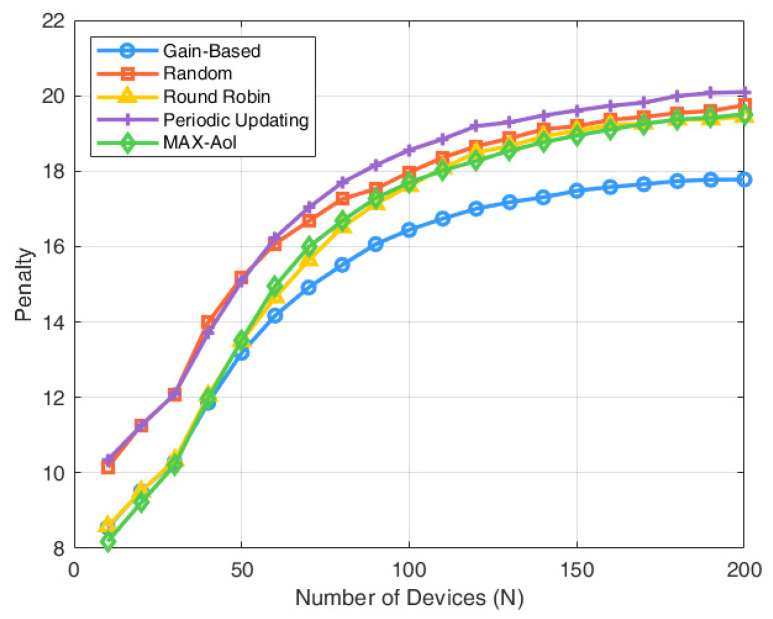
Penalty with number of devices.

As illustrated in Figure 4, the three strategies demonstrate nearly identical performance when N≤M, where the number of devices matches available channels, eliminating competition and enabling full scheduling capacity. However, as N increases, the performance of the random scheduling policy degrades significantly, since it selects at most M devices randomly for status updates, regardless of their urgency. In contrast, the gain-index-based policy makes more informed scheduling decisions by jointly considering both the freshness of information and the observed environmental state. On average, the gain-index strategy achieves a 10.75% improvement over random scheduling and a 5.71% improvement over round-robin scheduling. Compared to periodic updating, the gain-index strategy reduces the average penalty by 12.58%, and it further achieves a 5.29% reduction compared to the MAX-AoI policy. These results demonstrate the superior scalability and robustness of the proposed gain-index policy.

Considering the weight of the devices, the sum of the weights $\omega_{n}$ is normalized to 1. Some devices are assigned weights greater than 0, reflecting higher importance, while others may have weights closer to 0. In the weighted gain strategy, each device’s scheduling priority is determined by the product $\omega_{n}\cdot g_{n}\left(x,\delta ,\lambda \right)$, where $g_{n}\left(x,\delta ,\lambda \right)$ is the original gain index value that reflects the expected benefit of updating the device. This approach allows the scheduler to favor high-impact updates and leads to a consistent reduction in overall system penalty.

**Figure 5:**
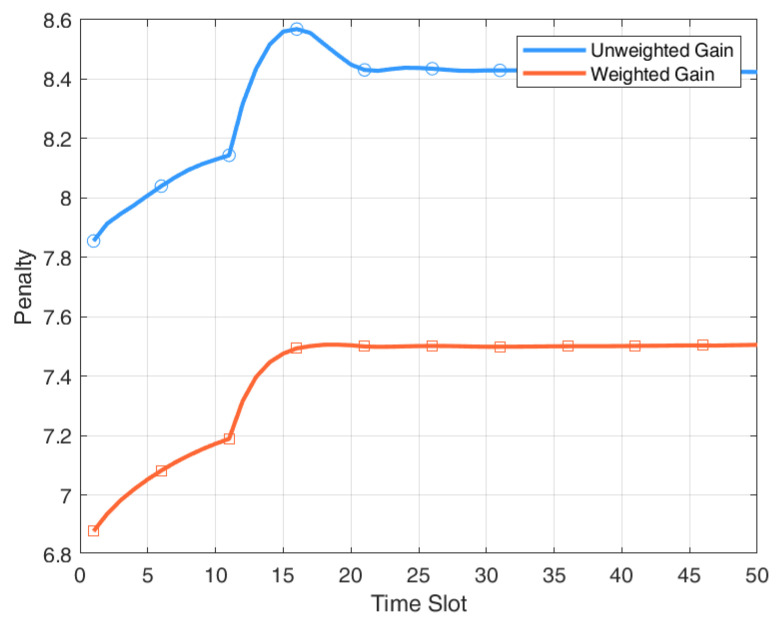
Weighted Gain vs. Unweighted Gain.

Figure 5 illustrates the evolution of system penalty over time under two scheduling strategies: weighted gain and unweighted gain, across T=50 time slots. The weighted gain strategy consistently achieves a lower system penalty than the unweighted gain strategy, with the performance gap especially evident in the early stages. On average, it reduces the penalty by 11.96% by prioritizing updates from high-impact devices, thereby improving information freshness and reducing decision errors in dynamic environments.

## 6. Limitations and Future Work

While the proposed entropy-based scheduling framework demonstrates significant improvements in minimizing system estimation loss, several limitations warrant further exploration. The current model assumes a static UAV position and does not consider trajectory optimization, which could further enhance performance in dynamic environments. In addition, the loss function relies on predefined misclassification costs that may not fully reflect real-world operational risks. The framework also adopts a centralized scheduling scheme with perfect knowledge assumptions, which may limit scalability in large-scale or partially observable IoT systems. Future work will focus on extending the framework to support joint UAV trajectory and scheduling optimization, learning-based estimation of penalties, and scalable distributed implementations. Furthermore, integrating the proposed approach with emerging paradigms such as integrated sensing and communication (ISAC) offers a promising direction for jointly optimizing sensing quality and information freshness under resource constraints.

## 7. Conclusions

In this paper, we proposed an L-conditional cross-entropy-based, age-aware scheduling strategy for UAV-assisted IoT data transmission. By incorporating the L-conditional cross-entropy between the true and estimated status as the penalty metric, our approach effectively captures the estimation degradation caused by outdated information, especially under asymmetric loss costs. We modeled the scheduling problem using a Restless Multi-Armed Bandit (RMAB) framework and introduced a net-gain-based index policy. By applying Lagrange relaxation and dual gradient updates, we derived an efficient gain function that guides the UAV in selecting the most valuable sources to update at each time step. Unlike traditional Whittle index approaches, the proposed strategy does not require indexability conditions, making it more widely applicable to real-world IoT monitoring tasks with heterogeneous sensor dynamics and cost structures. Simulation results demonstrated that the proposed method significantly outperforms baseline scheduling strategies such as round-robin and random selection, particularly under challenging conditions involving limited bandwidth and critical monitoring tasks such as fire detection, where information freshness is crucial.

## Figures and Tables

**Table 1:** Example of loss values.

True State (TS)\Estimated State (ES)	Normal (N)	Alert (A)	Fire (F)
Normal (N)	$L\left(N,N\right)=0$	$L\left(N,A\right)=20$	$L\left(N,F\right)=20$
Alert (A)	$L\left(A,N\right)=50$	$L\left(A,A\right)=0$	$L\left(A,F\right)=30$
Fire (F)	$L\left(F,N\right)=300$	$L\left(F,A\right)=100$	$L\left(F,F\right)=0$

## Data Availability

The original contributions presented in this study are included in the article. Further inquiries can be directed to the corresponding author.

## Appendix A

### Proof of Convergence of Dual Problem

To solve the relaxed scheduling problem in (15), we apply a dual decomposition approach with a Lagrange multiplier λ and iteratively update it using the following stochastic rule

(A1) $$\lambda_{t+1}=\max\left\{\lambda_{t}+\alpha_{t}\left(\sum_{n=1}^{N}u_{n}\left(t\right)-M\right),0\right\}=\prod_{\left[0,\infty \right)}\left[\lambda_{t}+\alpha_{t}\left(\sum_{n=1}^{N}u_{n}\left(t\right)-M\right)\right],$$

where $\alpha_{t}=\frac{c}{t}$,c is a constant, $u_{n}\left(t\right)\in \left\{0,1\right\}$ is the scheduling decision for device n at time t, and M is the total number of available communication channels. $\prod_{\left[0,\infty \right)}\left[\cdot \right]$ defines the projection operator onto the non-negative orthant, $\lambda_{t}$ is the dual variable associated with the long-term average constraint, and $\lambda^{*}$ is the optimal solution to the dual problem (15).

In what follows, we focus on establishing the convergence of the dual subgradient method applied to the dual optimization problem. Specifically, we analyze the sequence of dual variables generated by projected subgradient updates and study the behavior of the corresponding dual function values. By leveraging the concavity of the dual function and the basic iterate inequality from Lemma 2 in [31], we aim to demonstrate that the time-averaged dual function values asymptotically converge to the optimal dual value. As mentioned in the previous text, the dual function is defined as:

(A2) $$J\left(\lambda \right)=\underset{\pi \in \Pi }{\inf}L\left(\pi ,\lambda \right),$$

where

(A3) $$L\left(\pi ,\lambda \right)=\underset{T\to \infty }{\lim\sup}\sum_{n=1}^{N}E_{\pi }\left[\frac{1}{T}\sum_{t=0}^{T-1}\omega_{n}p_{n}\left(X_{n,t-\Delta \left(t\right)},\Delta \left(t\right)\right)+\lambda u_{n}\left(t\right)\right]-\lambda M,$$

This is the pointwise infimum of a family of affine functions in λ. Hence, $J\left(\lambda \right)$ is concave, and $-J\left(\lambda \right)$ is convex [32,33]. Let

(A4) $$g_{t}=\sum_{n=1}^{N}u_{n}\left(t\right)-M.$$

Since this quantity is the derivative of $L\left(\pi ,\lambda \right)$ with respect to λ, evaluated at a fixed policy π under $\lambda_{t}$, we have the following:

(A5) $$E\left[g_{t}\right]\in \partial J\left(\lambda_{t}\right).$$

That is, the expected value of $g_{t}$ belongs to the subdifferential of the dual function at $\lambda_{t}$. Therefore, $g_{t}$ is an unbiased estimator of a subgradient of $J\left(\lambda_{t}\right)$. Since each $u_{n}\left(t\right)\in \left\{0,1\right\}$, and at most M devices can be scheduled, we have the following:

(A6) $$\begin{array}{l} g_{t}=\sum_{n=1}^{N}u_{n}\left(t\right)-M\\ g_{t}\in \left[-M,N-M\right]\\ \left|g_{t}\right|\leq \max\left\{-M,N-M\right\}≜G \end{array},$$

Therefore $g_{t}$ is uniformly bounded, $E\left[g_{t}^{2}\right]<\infty$.

We choose the step-size $\alpha_{t}=\frac{c}{t},c=1$, which satisfies the following:

(A7) $$\begin{array}{l} \sum_{t=1}^{\infty }\frac{1}{t}=\infty \\ \sum_{t=1}^{\infty }\frac{1}{t^{2}}<\infty \end{array},$$

According to [31], for any λ>0, we have the following:

(A8) $${\left\|\lambda_{t+1}-\lambda \right\|}^{2}\leq {\left\|\lambda_{t}-\lambda \right\|}^{2}-2\alpha_{t}\left[J\left(\lambda \right)-J\left(\lambda_{t}\right)\right]+{\alpha_{t}}^{2}G^{2}.$$

Sum the expressions as follows:

(A9) $$\sum_{t=0}^{T-1}{\left\|\lambda_{t+1}-\lambda \right\|}^{2}\leq \sum_{t=0}^{T-1}{\left\|\lambda_{t}-\lambda \right\|}^{2}-\sum_{t=0}^{T-1}2\alpha_{t}\left[J\left(\lambda \right)-J\left(\lambda_{t}\right)\right]+\sum_{t=0}^{T-1}{\alpha_{t}}^{2}G^{2}$$

Let $\lambda =\lambda^{*}$,

(A10) $$\sum_{t=0}^{T-1}2\alpha_{t}\left[J\left(\lambda^{*}\right)-J\left(\lambda_{t}\right)\right]\leq {\left\|\lambda_{0}-\lambda^{*}\right\|}^{2}-{\left\|\lambda_{T}-\lambda^{*}\right\|}^{2}+\sum_{t=0}^{T-1}{\alpha_{t}}^{2}G^{2},$$

(A11) $$\sum_{t=0}^{T-1}\alpha_{t}\left[J\left(\lambda^{*}\right)-J\left(\lambda_{t}\right)\right]\leq \frac{{\left\|\lambda_{0}-\lambda^{*}\right\|}^{2}+G^{2}\sum_{t=0}^{T-1}{\alpha_{t}}^{2}}{2},$$

Divide both sides by $T\alpha_{t}$,

(A12) $$\frac{1}{T}\sum_{t=0}^{T-1}J\left(\lambda_{t}\right)\geq J\left(\lambda^{*}\right)-\frac{B_{T}}{T\alpha_{t}},$$

where $\frac{B_{T}}{T\alpha_{t}}=\frac{{\left\|\lambda_{0}-\lambda^{*}\right\|}^{2}}{2\alpha_{t}T}+\frac{TG^{2}{\alpha_{t}}^{2}}{2\alpha T_{t}}$, as T→∞, $\frac{B_{T}}{T\alpha_{t}}\to 0$ and we obtain the following:

(A13) $$\underset{T\to \infty }{\lim\inf}\frac{1}{T}\sum_{t=0}^{T-1}J\left(\lambda_{t}\right)\geq J\left(\lambda^{*}\right),$$

According to the dual problem (15), the feasible region of the dual variables is $D=\left[0,\infty \right)$, and the optimal solution is $\lambda^{*}=\arg\max_{\lambda \in D}J\left(\lambda_{t}\right)$, therefore, we have $J\left(\lambda^{*}\right):=\max_{\lambda \in D}J\left(\lambda_{t}\right)$ and $J\left(\lambda_{t}\right)\leq J\left(\lambda^{*}\right)$. By integrating Equation (A14), we derive the following:

(A14) $$\underset{T\to \infty }{\lim}\frac{1}{T}\sum_{t=0}^{T-1}J\left(\lambda_{t}\right)=J\left(\lambda^{*}\right),$$

The above result establishes that the time-averaged dual function values generated by the projected subgradient method converge to the optimal dual value.
